# Intratendinous Porcine‐Derived Type I Collagen Injections Result in Clinical and Radiographic Improvement of Rotator Cuff Tendinopathy 1 Year After Treatment

**DOI:** 10.1002/ars2.70114

**Published:** 2026-10-05

**Authors:** Pietro Simone Randelli, Antonio Muzio, Carlo Stoppani, Chiara Fossati, Mauro Battista Gallazzi, Alessandra Menon

**Affiliations:** ^1^ Laboratory of Applied Biomechanics, Department of Biomedical Sciences for Health Università degli Studi di Milano Milan Italy; ^2^ U.O.C. 1° Clinica Ortopedica ASST G. Pini‐CTO Milan Italy; ^3^ Research Center for Adult and Pediatric Rheumatic Diseases (RECAP‐RD), Department of Biomedical Sciences for Health Università degli Studi di Milano Milan Italy; ^4^ U.O.C. Week Surgery ASST Gaetano Pini‐CTO Milan Italy; ^5^ Department of Radiology ASST Gaetano Pini‐CTO Milan Italy

## Abstract

**Purpose:**

To evaluate the clinical and radiological effectiveness of porcine‐derived type I collagen injections in patients with symptomatic rotator cuff tendinopathy.

**Methods:**

In this monocentric pilot study conducted between 2022 and 2025, adults with clinically and radiologically confirmed rotator cuff tendinopathy received 3 ultrasound‐guided intratendinous injections at 2‐week intervals and were followed for 12 months. A power analysis determined that 24 patients were required to detect a 13‐point improvement in the Constant‐Murley Score (CMS) at 3 months. The primary outcome was the achievement of a ≥13‐point increase in the CMS at 3 months. Secondary outcomes included functional scores, pain intensity, magnetic resonance imaging findings, and analgesic consumption.

**Results:**

Twenty‐four patients were enrolled, and 22 completed 12‐month follow‐up. At 3 months, 8 out of 23 patients (35%) achieved the predefined 13‐point CMS as minimal clinically important difference. Mean CMS improved from 62.4 at baseline to 72.2 at 3 months and 78.7 at 12 months (*P* < .001). The American Shoulder and Elbow Surgeons score improved from 47.3 at baseline to 68.5 at 3 months and 82.2 at 12 months. The Simple Shoulder Test improved from 8.8 to 9.7 at 3 months and 11.3 at 12 months. Pain scores decreased from 5.8 at baseline to 3.5 at 3 months and 2.1 at 12 months. Magnetic resonance imaging at 12 months showed improved tendon quality in 7 out of 20 evaluated patients (35%).

**Conclusions:**

Intratendinous porcine‐derived type I collagen injections were associated with progressive improvements in shoulder function and pain over 12 months in this single‐arm pilot study. Although the predefined minimal clinically important difference at 3 months was reached by a proportion of patients, functional scores showed continued improvement throughout follow‐up. Magnetic resonance imaging findings showed improved tendon quality in part of the cohort, and no serious adverse events were recorded.

**Level of Evidence:**

Level IV, prospective therapeutic case series.

## Graphical abstract

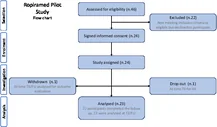

Rotator cuff (RC) diseases are among the most common musculoskeletal conditions, after lower back and knee pain.^1^ The prevalence of partial‐thickness RC tears is estimated to range from 13% to 37% and is expected to rise with the aging of the population, given the positive correlation between age and RC disease.^2^ Most cases are attributed to RC tendinopathy or partial‐ and full‐thickness RC tears.^3^, ^4^ Most cases are related to RC tendinopathy and degenerative tendon changes characterized by extracellular matrix disorganization, collagen remodeling, hypercellularity, and neovascularization, which impair tendon mechanical properties and contribute to chronic pain.^5^

The optimal management of RC diseases remains controversial and depends on tear characteristics, symptom severity, functional impairment, and patient expectations.^6^ In RC tendinopathy and partial‐thickness tears, nonoperative management is generally considered first line. This approach typically includes oral pharmacological therapy, structured physical rehabilitation, and temporary activity modification.^7^ Therapeutic exercise is recognized as the most effective nonoperative option, although the optimal protocol remains unclear, and physiotherapy alone may have limited impact on disease progression. These limitations have prompted interest in biological therapies. Biologic strategies have therefore been investigated to enhance tendon healing. Platelet‐rich plasma is widely used but shows inconsistent clinical efficacy, whereas cell‐based therapies remain limited by regulatory and procedural constraints.^8^, ^9^, ^10^ Recently, in vitro studies by Randelli et al. evaluated the effects of a porcine‐derived injectable type I collagen on tenocyte activity, showing predominantly mechanical effects with significant morphofunctional modifications.^11^, ^12^

Building on these preclinical findings, the purpose of this study was to evaluate the clinical and radiological effectiveness of porcine‐derived type I collagen injections in patients with symptomatic RC tendinopathy. We hypothesized that treatment would result in a clinically meaningful improvement, specifically an increase of at least 13 points in the Constant‐Murley Score (CMS)^13^ at 3‐month follow‐up compared with baseline.

## METHODS

### Study Design

The study is a prospective pilot investigation conducted at the ASST Gaetano Pini‐CTO, Milan, Italy. The study protocol was approved by the Regional Ethical Committee (Comitato Etico Milano Area 2, 427_2022, April 12, 2022; amendment 1, 159_2023, February 14, 2023), registered at ClinicalTrials.gov (NCT05489484; August 8, 2022), and carried out in accordance with the Declaration of Helsinki and Good Clinical Practice guidelines. All patients provided written informed consent for imaging examinations and data management prior to the enrollment.

Consecutive patients referring to the investigation center for symptomatic RC tendinopathy or partial tears (types A and B according to Snyder classification),^14^ confirmed by clinical examination and magnetic resonance imaging (MRI), were assessed for eligibility and enrolled between October 2022 and January 2025. Inclusion criteria were age ≥18 years, symptom duration ≥3 months, and a CMS between 40 and 75 points. Exclusion criteria were full‐thickness RC tears (Snyder type C), shoulder instability, adhesive capsulitis, previous hyaluronic acid or corticosteroid injections within the preceding 3 months, diabetes mellitus, uncontrolled thyroid disease, coagulopathy, chronic immunosuppressive therapy, known allergy to porcine collagen, and pregnancy or breastfeeding.

All patients received a cycle of 3 ultrasound‐guided intratendinous infiltrations of collagen administered at 2‐week intervals. The injections were performed by 2 experienced radiologists.

The 3‐injection protocol was selected on the base of previously published clinical studies investigating collagen‐based treatments for RC pathology, in which multiple administrations were associated with progressive clinical improvement and biological effects.

At the time of the first injection, patients were instructed to manage pain exclusively with paracetamol (1000 mg) or celecoxib (200 mg), taken as needed. No other analgesic or anti‐inflammatory medications were permitted during the study period. Analgesic consumption was prospectively recorded by patients in a daily diary and entered into the electronic Case Report Form (e‐CRF) at each follow‐up visit.

At the same visit, all patients were provided with a standardized home‐based shoulder exercise program consisting of mobility and strengthening exercises. The program was to be performed independently for approximately 30 minutes, twice weekly, during the first 3 months after the initial injection.

Supervised physiotherapy sessions were not permitted during the study period to ensure treatment consistency across participants. No additional infiltrative treatments (e.g., corticosteroids, hyaluronic acid, or biologic injections) were allowed during follow‐up.

### Follow‐Up

Each participant was followed for 12 months. Clinical evaluations were performed at baseline; 2‐ and 4‐week; and 3‐, 6‐, and 12‐month follow‐up. Data were recorded in e‐CRF.

All clinical outcome measures were prospectively collected at each scheduled in‐person office visit by the same fellowship‐trained orthopaedic surgeon (Antonio Muzio), expert in the administration of standardized shoulder outcome instruments and not involved in the injection procedure. Data were entered into the e‐CRF immediately after each evaluation. The database was reviewed for completeness and accuracy prior to statistical analysis.

The primary outcome was treatment efficacy of the collagen‐based medical device MD‐Shoulder (Guna S.p.a. Milan, Italy) defined as achieving an increase of at least 13 points in the CMS at 3 months compared with baseline. The previously reported minimal clinically important difference (MCID) values for the CMS in RC‐related shoulder disorders range approximately between 10 and 15 points.^15^, ^16^ The value of 13 points was selected a priori as an intermediate threshold within this reported range.

The CMS was assessed according to the original description by Constant and Murley. Pain (15 points) and activities of daily living (20 points) were obtained through structured patient interview. Range of motion (ROM) (40 points), defined as active forward elevation, abduction, external rotation with the arm at the side, and internal rotation, was measured using a standard goniometer with the patient in the standing position. Strength (25 points) was assessed in 90° of abduction in the scapular plane using a handheld dynamometer.

The secondary outcomes included CMS at 6‐ and 12‐month follow‐up compared with baseline; pain intensity assessed by Numeric Rating Scale^17^ at 2‐ and 4‐week and 3‐, 6‐, and 12‐month follow‐up; functional outcomes assessed by the American Shoulder and Elbow Surgeons score^18^ and the Simple Shoulder Test,^19^ as well as ROM at 3‐, 6‐, and 12‐month follow‐up; RC integrity at 12 months evaluated by 1.5 T MRI using the predefined 4‐grade classification system, analgesic consumption during follow‐up, and assessment of adverse events.

Adverse events were prospectively monitored at each follow‐up visit and recorded in the e‐CRF. Patients were specifically asked about local and systemic complications related to the injection procedure, and any spontaneous reports were documented. Adverse events were classified according to severity and relation to the treatment.

### Clinical and Radiological Assessment

Patients with supraspinatus tendinopathies and/or partial tears, documented by high‐field MRI, were enrolled in the study. A second MRI evaluation was conducted at 12 months after the enrollment, during which all patients underwent MRI at the ASST G. Pini‐CTO using a 1.5 T scanner. All enrolled patients had isolated partial lesions of the supraspinatus tendon, either articular or bursal (types A and B according to Snyder),^14^ assessed by both an orthopaedic surgeon (Antonio Muzio) and a radiology specialist (M.B.G.). Fatty infiltration was assessed according to the Fuchs classification.^20^ A simplified 4‐grade MRI‐based classification system was used to evaluate supraspinatus tendinopathy, adapted from the scheme originally proposed for Achilles tendon disorders by Pomeranz^21^ and later validated by Movin et al.^22^ Grade 1 was defined as normal tendon with homogeneous low signal intensity on both T1‐ and fluid‐sensitive weighted sequences, with preserved fibrillar architecture. A minimal amount of peritendinous bursal fluid may be present and is considered within physiological limits. Grade 2 was defined as early tendon degeneration characterized by focal signal changes visible on T1‐weighted sequences without tendon thickening. Tendon size and structure are preserved. Fluid‐sensitive sequences show minimal or no abnormal signal. Grade 3 was defined as tendon matrix thickening and degeneration, with an increased tendon thickness and disorganized internal matrix with signal changes on both T1‐ and fluid‐sensitive sequences, consistent with moderate degeneration. The tendon shows loss of normal architecture but remains continuous. Grade 4 was defined as severe degeneration with tendon tear, characterized by full‐thickness or high‐grade partial tear of the tendon, with structural discontinuity, tendon stump retraction, and high signal on fluid‐sensitive sequences in the defect and surrounding fluid, often associated with marked degeneration of the remaining tendon.

### Statistical Analysis

This is a 1‐sample pilot study. It has been assumed that the CMS at baseline was 65.0, and based on eligibility criteria, this value was included in the range from 50 to 80 so that the expected standard deviation would have been 7.5. At the end of the treatment (3 months), an average increase of 13 in the score was expected, as well as a standard deviation of differences equal to 20.

Given the above assumptions, a sample of 24 subjects should have reached a 0.862 power to detect the expected difference with a Student's *t* test for paired data. In the case of nonapplicability of the Student's *t* test, the use of the Wilcoxon's nonparametric equivalent would have led to a 0.828 power.

Based on the data observed retrospectively in the study, a cohort‐derived MCID was also calculated using the half‐standard deviation method. Using a Monte Carlo bootstrap simulation based on the 23 patients who completed the primary outcome assessment, the estimated MCID was 6.52 points. This cohort‐derived MCID was considered exploratory and specific to the present sample.

Data for the secondary outcomes have been analyzed using repeated‐measures analysis of variance with Bonferroni correction for multiple comparisons.

Statistical significance was set at *P* < .05, unless multiple comparisons were to be assessed. Analyses were performed by an independent statistician. Stata/SE 17.0 has been used for statistical calculations.

## RESULTS

A total of 46 patients were screened for eligibility. Twenty‐four patients were enrolled in the study, of whom 23 completed the 3‐month follow‐up (primary end‐point) and 22 completed the 12‐month follow‐up (Figure 1). Baseline demographic and clinical characteristics of the study population are summarized in Table 1.

**FIGURE 1:**
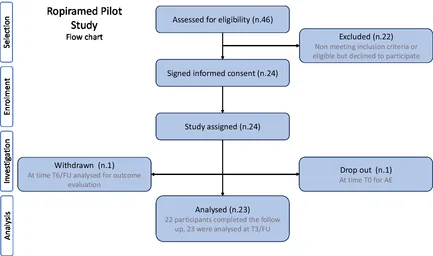
Flow diagram of the study. (AE, adverse event; FU, follow‐up.)

**TABLE 1:** Patients’ Demographics at Baseline

No. of patients	24
Sex	
Female	16 (66.7)
Male	8 (33.3)
Age at follow‐up, yr	53.18 ± 12.43
Dominant side treated	
No	6 (25)
Yes	18 (75)
Smoking	
Nonsmoker	19 (79.2)
Smoker	5 (20.8)
Manual worker	
No	16 (66.7)
Yes	8 (33.3)
Sport	
No	11 (45.8)
Yes	13 (54.2)
BMI	24.86 ± 4.31

*Note*: Data are reported as mean ± SD or number of patients (percentage).

BMI, body mass index; No., number; SD, standard deviation; yr, years.

The primary outcome, defined as an increase of at least 13 points in the CMS at 3 months, was successfully achieved by 8 patients among the 23 analyzed (34.8%; 95% confidence interval: 16.4‐57.3).

Using the post hoc cohort‐derived MCID calculated with the half‐standard deviation method, the estimated threshold was 6.52 points. Eighteen of 23 patients (78.3%) exceeded this threshold.

All clinical outcome scores (CMS, Numeric Rating Scale, American Shoulder and Elbow Surgeons, and Simple Shoulder Test) showed a progressive improvement over time, with statistically significant changes observed from 3 months onward for most outcomes and further gains at 6 and 12 months (Figures 2, 3, 4, 5).

**FIGURE 2:**
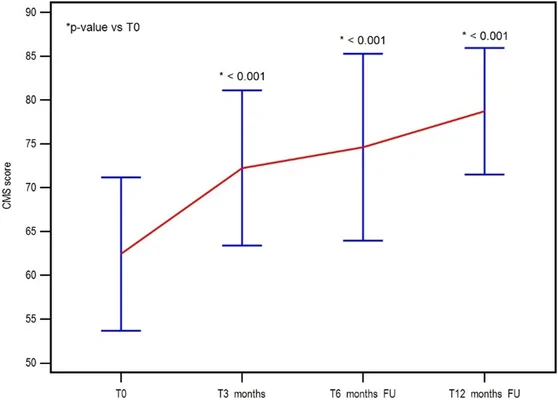
CMS values at baseline (T0) and at 3‐ (T3), 6‐ (T6 FU), and 12‐month follow‐up (T12 FU). Vertical bars indicate ±1 standard deviation. (CMS, Constant‐Murley Score; FU, follow‐up.)

**FIGURE 3:**
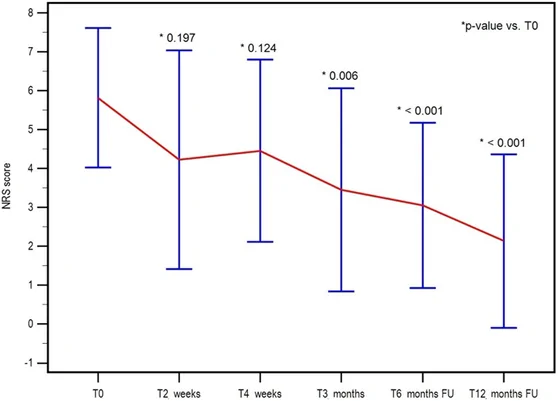
NRS values at baseline (T0) and at 3‐ (T3), 6‐ (T6 FU), and 12‐month follow‐up (T12 FU). Vertical bars indicate ±1 standard deviation. (FU, follow‐up; NRS, Numeric Rating Scale.)

**FIGURE 4:**
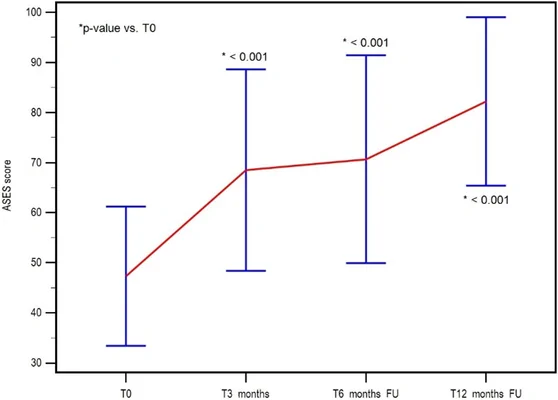
ASES score values at baseline (T0) and at 3‐ (T3), 6‐ (T6 FU), and 12‐month follow‐up (T12 FU). Vertical bars indicate ±1 standard deviation. (ASES, Assessment Shoulder and Elbow Surgeons; FU, follow‐up.)

**FIGURE 5:**
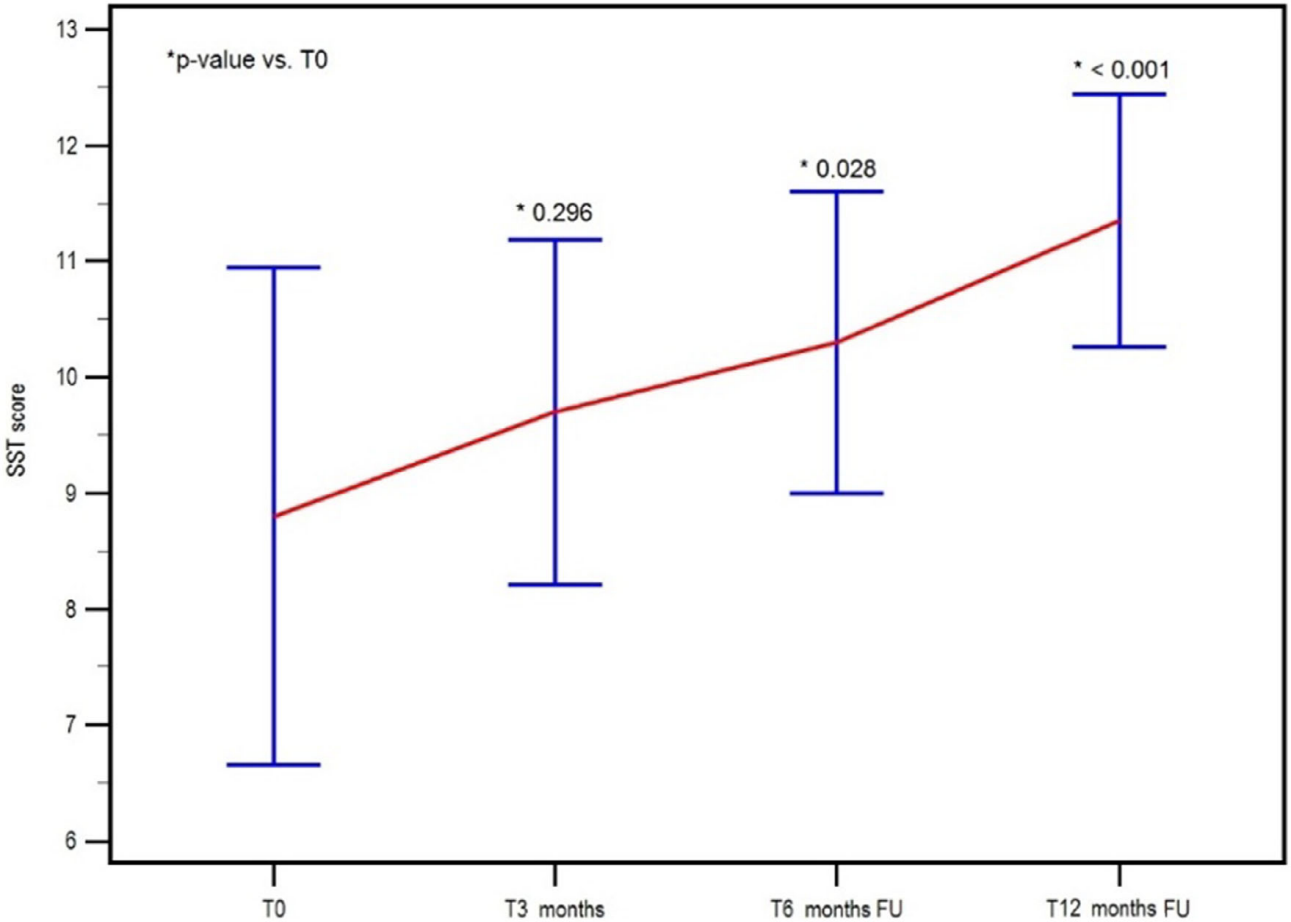
SST values at baseline (T0) and at 3‐ (T3), 6‐ (T6 FU), and 12‐month follow‐up (T12 FU). Vertical bars indicate ±1 standard deviation. (FU, follow‐up; SST, Simple Shoulder Test.)

No statistically significant changes were observed in ROM parameters (flexion, external rotation, internal rotation, and abduction) at any follow‐up time point compared with baseline (all *P* > .05). A comprehensive summary of clinical and functional outcomes across all time points is reported in Table 2.

**TABLE 2:** Summary of the Clinical and Functional Results at Primary and at the Follow‐Up Time Points*

**Outcome**	**Baseline**	**3 mo**	**6 mo**	**12 mo**	***P*** **Value** †	***P*** **Value** ‡	***P*** **Value** §
CMS	62.2 (1.75); n = 24	72.8 (1.90); n = 23	74.6 (2.27); n = 22	78.7 (1.54); n = 22	<.001	<.001	<.001
	58.6‐65.8 (44.6‐73.3)	68.9‐76.8 (52.1‐89.4)	69.9‐79.3 (49.7‐94.0)	75.5‐81.9 (64.5‐94.8)			
NRS	6.0 (0.36); n = 24	3.4 (0.53); n = 23	3.0 (0.45); n = 22	2.1 (0.48); n = 22	.006	<.001	<.001
	5.2‐6.7 (2.0‐8.0)	2.3‐4.5 (0.0‐10.0)	2.1‐4.0 (0.0‐7.0)	1.1‐3.1 (0.0‐7.0)			
ASES	45.2 (3.27); n = 24	69.1 (4.14); n = 23	70.7 (4.42); n = 22	82.2 (3.59); n = 22	<.001	<.001	<.001
	38.5‐52.0 (4.0‐78.3)	60.5‐77.7 (21.6‐95.0)	61.5‐79.9 (35.0‐100.0)	74.8‐89.7 (43.3‐100.0)			
SST	8.5 (0.47); n = 23	9.9 (0.32); n = 22	10.4 (0.29); n = 21	11.4 (0.22); n = 22	.296	.028	<.001
	7.5‐9.5 (3.0‐12.0)	9.2‐10.5 (7.0‐12.0)	9.8‐11.0 (8.0‐12.0)	10.9‐11.9 (8.0‐12.0)			
ROM FLE	175.2 (1.89); n = 24	177.6 (1.60); n = 23	174.5 (2.79); n = 22	175.5 (4.09); n = 22	>.999	>.999	>.999
	171.3‐179.1 (140‐180)	174.3‐180.9 (145‐180)	168.7‐180.3 (130‐180)	166.9‐184.0 (90‐180)			
ROM FLE	175.2 (1.89); n = 24	177.6 (1.60); n = 23	174.5 (2.79); n = 22	175.5 (4.09); n = 22	>.999	>.999	>.999
	171.3‐179.1 (140‐180)	174.3‐180.9 (145‐180)	168.7‐180.3 (130‐180)	166.9‐184.0 (90‐180)			
ROM EXT	79.2 (3.26); n = 24	86.1 (2.01); n = 23	83.9 (2.55); n = 22	85.9 (1.02); n = 22	.956	>.999	>.999
	72.4‐85.9 (45‐90)	81.9‐90.3 (45‐90)	78.6‐89.2 (45‐90)	83.8‐88.0 (80‐90)			
ROM INT	76.7 (3.33); n = 24	84.8 (1.35); n = 23	79.5 (3.66); n = 22	85.2 (2.17); n = 22	.082	>.999	.149
	69.8‐83.6 (40‐90)	82.0‐87.6 (70‐90)	71.9‐87.2 (45‐90)	80.7‐89.7 (45‐90)			
ROM ABD	144.4 (3.45); n = 24	147.2 (2.61); n = 23	149.1 (0.91); n = 22	150.0 (0.00); *n* = 22	>.999	>.999	.700
	137.2‐151.5 (90‐150)	141.8‐152.6 (90‐150)	147.2‐151.0 (130‐150)	150.0‐150.0 (150‐150)			

ABD, abduction; ASES, American Shoulder and Elbow Surgeons;^18^ CMS, Constant‐Murley Score;^13^ EXT, external rotation; FLE, flexion; INT, internal rotation; mo, month; NRS, Numeric Rating Scale;^17^ ROM, range of motion; SST, Simple Shoulder Test.^19^

* Data are reported as mean (standard error of the mean) and sample size (n) for each time point. The second line reports 95% confidence intervals, with ranges (minimum‐maximum) in parentheses.

† Significance of the within‐group difference at 3‐months follow‐up.

‡ Significance of the within‐group difference at 6‐month follow‐up.

§ Significance of the within‐group difference at 12‐month follow‐up.

Of the 22 patients who completed the 12‐month follow‐up, 20 underwent MRI evaluation. Improvement in tendon quality, defined as a decrease of at least 1 grade according to the Movin classification, was observed in 7 patients (35%), whereas 11 (55%) remained stable and 2 (10%) showed worsening. No progression of fatty degeneration was observed. Detailed MRI findings are reported in Table 3.

**TABLE 3:** MRI Findings at 12‐mo Follow‐Up

	**Tendon Quality (Movin Classification)**	**Fatty Degeneration (Fuchs Classification)**
No. of patients improved at 12‐mo FU	7 (35)	7 (35)
No. of patients stable at 12‐mo FU	11 (55)	13 (65)
No. of patients worsened at 12‐mo FU	2 (10)	0 (0)

*Note*: Data are reported as number of patients (percentage). Tendon quality was assessed using the Movin^22^ classification, whereas fatty degeneration was evaluated according to the Fuchs^20^ classification. Improvement, stability, and worsening were defined as a decrease, no change, and an increase of at least 1 grade compared with baseline MRI, respectively.

FU, follow‐up; MRI, magnetic resonance imaging; mo, month; No., number.

Occasional analgesic use was reported by 13 of 23 patients (56.5%; 95% confidence interval: 34.5‐76.8).

The treatment was well tolerated. One mild cutaneous reaction occurred 48 hours after the first injection and resolved spontaneously without medical intervention. No further adverse events were reported.

## DISCUSSION

The most important finding of this prospective pilot study is that intratendinous injections of porcine‐derived type I collagen were associated with progressive clinical improvement in patients with RC tendinopathy.

The primary outcome, defined as a +13‐point improvement in CMS at 3 months, was not achieved because 8 out of 23 analyzed patients (35%) reached this level of improvement. However, CMS showed a progressive increase over time, from +10 points at 3 month to +16 points at 12‐month follow‐up. It should be emphasized that a +13‐point CMS improvement represents a clinically meaningful and ambitious target in this patient population. A post hoc cohort‐derived MCID analysis using the half‐standard deviation method yielded a threshold of 6.52 points, which was exceeded by 78.3% of patients; however, this value should be interpreted cautiously because it is specific to the present cohort and was not prespecified in the study protocol. The +16‐point improvement observed at 12 months supports the clinical relevance of the treatment response and may be consistent with the biological timeline of collagenous tissue repair and tendon remodeling;^23^, ^24^ however, causality cannot be established in the absence of a control group. This pattern differs from commonly used injections such as corticosteroids, which may provide faster pain relief but do not aim to promote structural tendon remodeling. In this context, the progressive clinical and imaging improvements observed in the present study may reflect a biologically oriented treatment strategy targeting tendon healing rather than short‐term symptom control.

From a clinical perspective, even a +7‐point improvement in CMS may be considered satisfactory in patients with RC tendinopathy.^25^

Additionally, although the primary outcome was not achieved, the collected data showed meaningful improvements in CMS scores, comparable to the results by Corrado et al.^26^ and more favorable than the outcomes reported by Chae et al.,^27^ where a single infiltration of atelocollagen, a collagen‐based biomaterial different from the porcine‐derived type I collagen used in the present study, failed to produce clinical benefit at the 2‐month follow‐up. Patients who completed the full course of collagen injections showed progressive improvements in shoulder function, as reflected by American Shoulder and Elbow Surgeons and Simple Shoulder Test scores. These findings align with previously published results by Kim et al.^23^ and Buda et al.^24^

Pain reduction was observed at 3‐, 6‐, and 12‐month follow‐up, consistent with earlier studies reporting analgesic effects of collagen‐based injections.

A recent systematic review of studies investigating collagen injections for RC disorders (including tendinopathy and partial tears) confirmed their beneficial effects on both shoulder function and pain relief.^28^ These outcomes were observed despite heterogeneity in injection techniques (intratendinous, subacromial bursa, or tendon‐bone interface) and treatment protocols (ranging from 1 to 4 infiltrations).

However, intratendinous administration was associated with greater immediate postprocedural pain, and 1 case of lesion progression was reported.^29^

The decision to perform intratendinous rather than peritendinous injections was based on biological principles of tendon healing. As highlighted by Sripathi and Agrawal, extrinsic healing leads to a disorganized, adhesion‐prone collagen matrix, whereas intrinsic healing—driven by tenocytes—produces better‐organized and mechanically superior tissue.^30^

Delivering the collagen device directly into the tendon was therefore chosen to preferentially stimulate intrinsic repair mechanisms, targeting the core of the lesion and promoting a more effective regenerative response.

Godek et al.^29^ compared the efficacy of various infiltrative approaches (collagen alone, platelet‐rich plasma, and combination of collagen and platelet‐rich plasma) for the treatment of partial‐thickness RC tears. Pain measured with the Numeric Rating Scale decreased significantly during follow‐up, from approximately 5 to 6 points at baseline to about 1 to 2 points at 24 weeks, without significant differences between treatment groups (*P* = .870). These results are comparable to the progressive reduction in pain observed in the present study.

At final MRI follow‐up, the percentage of patients showing improved tendon quality and reduced lesion size was consistent with the findings of Aicale et al.^28^ These results are consistent with a potential effect on tendon healing through the remodeling of the extracellular matrix.

No statistically significant changes were observed in ROM parameters, which is consistent with the typical clinical presentation of RC tendinopathy, a condition that typically does not involve significant joint mobility limitations.

The treatment was well tolerated; only a single adverse event was reported, consisting of a mild cutaneous reaction occurring approximately 48 hours after the first injection, which did not require medical intervention. These findings support the good safety profile of the treatment, particularly in light of the review by Aicale et al.,^28^ which reported no adverse events associated with collagen administration, as well as the absence of adverse reactions to MD‐Tissue in the clinical study by Randelli et al.^31^

Importantly, clinical and MRI improvements in this cohort were progressive over time, with measurable changes already present at early follow‐up and 12‐month evaluation primarily confirming persistence and structural stability rather than delayed onset of effect. This temporal pattern is clinically relevant in patients with persistent symptoms despite prior conservative treatment and suggests a sustained biological response after intratendinous collagen administration.

### Limitations

This study has some limitations. It has a small sample size, which prohibits subgroup analysis to analyze sex‐based differences. Furthermore, the absence of a control group does not allow definitive conclusions regarding the comparative efficacy of collagen injections versus standard nonoperative treatment or the natural history of the condition. Moreover, the absence of a predefined adjustment for potential dropouts led to a reduction in statistical power.

## CONCLUSIONS

Intratendinous porcine‐derived type I collagen injections were associated with progressive improvements in shoulder function and pain over 12 months in this single‐arm pilot study. Although the predefined MCID at 3 months was reached by a proportion of patients, functional scores showed continued improvement throughout follow‐up. MRI findings showed improved tendon quality in part of the cohort, and no serious adverse events were recorded.

## DISCLOSURES

The authors (P.S.R., C.F., Alessandra Menon) declare the following financial interests/personal relationships which may be considered as potential competing interests: P.S.R. reports a relationship with Medacta International that includes consulting or advisory and MicroPort Orthopedics that includes consulting or advisory. C.F. reports administrative support, article publishing charges, and equipment, drugs, or supplies provided by Guna. Alessandra Menon reports a relationship with Lipogems International that includes consulting or advisory and Adler Ortho that includes consulting or advisory. The other authors (Antonio Muzio, C.S., M.B.G.) declare that they have no known competing financial interests or personal relationships that could have appeared to influence the work reported in this article.

## FUNDING

Open access publishing facilitated by Azienda Socio Sanitaria Territoriale Centro Specialistico Ortopedico Traumatologico Gaetano Pini CTO, as part of the Wiley – SBBL agreement.
